# Correlation between heparanase gene polymorphism and susceptibility to endometrial cancer

**DOI:** 10.1002/mgg3.1257

**Published:** 2020-09-01

**Authors:** Hanyu Cao, Shuo Yang, Xiuzhang Yu, Mingrong Xi

**Affiliations:** ^1^ Department of Gynecology and Obstetrics West China Second University Hospital Sichuan University Chengdu China; ^2^ Key Laboratory of Obstetrics, Gynecology, Pediatric Disease, and Birth Defects Ministry of Education West China Second University Hospital Chengdu China; ^3^ Key Laboratory of Birth Defects and Related Diseases of Women and Children Ministry of Education Chengdu China

**Keywords:** endometrial cancer, heparanase, single‐nucleotide polymorphism

## Abstract

**Background:**

Endometrial cancer is one of the three most common malignancies in the female genital tract. Previous studies have demonstrated the association between heparanase (*HPSE*, OMIM 604,724) single‐nucleotide polymorphism (SNP) and cancer risk in several cancers. However, its role in endometrial cancer remains unclear. The present study investigated the effects of *HPSE* SNPs on the susceptibility and clinicopathological parameters in patients with endometrial cancer.

**Methods:**

*HPSE* SNPs of rs4693608 (G > A) and rs4364254 (C > T) were analyzed using polymerase chain reaction‐restriction fragment length polymorphism (PCR‐RFLP) assay in 270 endometrial cancer patients and 320 healthy controls.

**Results:**

The investigation indicated that the *HPSE* SNP rs4693608 with GG showed a protective effect from EC in both codominant (adjusted OR = 0.41, 95%CI = 0.21–0.81, *p* = .026) and recessive models (adjusted OR = 0.43, 95%CI = 0.22–0.82, *p* = .0076). No significant differences were found in the incidences of EC patients with the rs4364254 polymorphisms compared to controls. Moreover, a significantly increased distribution of A/A (rs4693608) was observed in patients with grade ≥ 2 (*p* = .03) and in patients with positive cervical invasion (*p* = .042) while patients with T/C (rs4364254) had lower tumor grade.

**Conclusion:**

Our study suggested that *HPSE* SNP of rs4693608 correlated strongly with susceptibility to EC, and *HPSE* SNPs might be a potential biomarker for prognosis of endometrial cancer.

## Graphical abstract

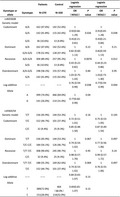

## 1 INTRODUCTION

Endometrial cancer (EC) is the most common gynecologic malignancy, accounting for 4.8% of all cancers diagnosed in women (Ferlay et al., 2015). There were around 60,000 new cases and 10,000 deaths each year in the United States and its incidence and mortality keeps on rising (Siegel, Miller, & Jemal, 2015, 2018). In China, the incidence of EC has surpassed cervical cancer and ranked first in gynecological cancers in developed cities since 2008 with the widescale screening of cervical cancer (Wei, 2013). At present, surgery remains the mainstay of therapy for EC and the adjuvant treatment followed based on the final histological results. However, there are a series of problems to be solved urgently, such as the tolerance of operation for senile patient and the fertility preservation for young patients as well as the high recurrence rate in advanced stages. In this sense, it is imperative to explore novel pathways and therapies for endometrial cancer treatment at the genetic level.

Heparanase (*HPSE*, OMIM 604,724) is the only known endo‐β‐glucuronidase in mammals. It was first identified in the late 1980s, when two independent groups demonstrated its enzymatic activity of degrading heparan sulfate (HS) chains in B16 melanoma cells and in T lymphoma cells (Masola, Zaza, Gambaro, Franchi, & Onisto, 2020; Nakajima, Irimura, Ferrante, Ferrante, & Nicolson, 1983; Vlodavsky, Fuks, Bar‐Ner, Ariav, & Schirrmacher, 1983). After the cloning of a single human heparanase cDNA in 1999 and the presence of derivative genetic tools, researchers began to accept the notion that this enzyme activity toward HS affects various biological activities including remodeling of the ECM barrier and regulating of HS‐linked cytokines and growth factors, contributing to tumor angiogenesis and metastasis (Barash et al., 2010; Iozzo & Sanderson, 2011; Sanderson, Yang, Suva, & Kelly, 2004; Vlodavsky & Friedmann, 2001). Previous studies showed high *HPSE* expression in nearly all human carcinomas examined including renal (Mikami et al., 2008), thyroid (Matos et al., 2015), hepatocellular (Chen, Dang, Luo, Feng, & Tang, 2008), lung (Fernandes et al., 2014), breast (Gawthorpe et al., 2014), ovarian (Davidson et al., 2007), and endometrial cancer (Inamine et al., 2008). Moreover, the mediating role of *HPSE* in the tumor microenvironment was also identified and *HPSE* has been considered as a potential anticancer target tested in clinical trials (Gutter‐Kapon et al., 2016; Rivara, Milazzo, & Giannini, 2016).

The *HPSE* located on the human chromosome 4q21.3 and expressed two mRNA species of 5 kb form and 1.7 kb form, respectively (Dong, Kukula, Toyoshima, & Nakajima, 2000). Various studies have evaluated the genetic frequencies of *HPSE* polymorphisms in different cancers and diseases. However, its role in endometrial cancer remains somehow unclear due to scarce evidence. In this study, we examined the association between two single‐nucleotide polymorphisms (SNPs) rs4693608 (G > A) and rs4364254 (C > T) and susceptibility to endometrial cancer.

## 2 MATERIAL AND METHODS

### 2.1 Study population

A total of 610 patients (270 EC patients and 340 age‐matched controls) from our hospital between June 2008 and June 2014 were recruited. The diagnosis of endometrial cancer was proven by pathologists using histopathological methods. The control group consisted of healthy women who underwent routine gynecological examinations in our outpatient department with no abnormalities. Relevant information was collected including age at diagnosis, body mass index (BMI), parity, family history of cancer, menopausal state, stage, grade, histology, ER/PR, myometrial invasion, cervical invasion, parametrial invasion, lymph node metastasis, lymphovascular space invasion. Staging was based on the International Federation of Gynecology and Obstetrics (FIGO) 2009 classification system.

### 2.2 Ethics statement

This research project was approved by the Ethical Committee of West China Second University Hospital of Sichuan University and was performed in line with the Declaration of Helsinki principles. All patients and healthy controls provided written consent.

### 2.3 DNA extraction and genotyping

Genomic DNA was isolated from peripheral blood following the instructions of the whole blood genomic DNA Extraction Kit (Tiangen, Beijing). DNA samples were stored at −20°C. The NanoDrop lite Spectrophotometer (Thermo Scientific) was used for detecting DNA concentrations. The SNPs of rs4693608 (G > A) and rs4364254 (C > T) were genotyped by a PCR‐RFLP (polymerase chain reaction‐restriction fragment length polymorphism) assay using the forward primer, 5′‐TTTCCTCTTGCCATCATGGG‐3′, the reverse primer, 5′‐TGACCAGGGTGGATTTTTTC‐3′ for rs4693608 (NT_016354.17 (intron 3)), and the forward primer, 5′‐TACCCACTTCAGCTTCCCAAA‐3′, the reverse primer, 5′‐GTCAAGAATGATCAGAGTTTAAGTATTCTTGGATAT‐3′ for rs4364254 (NT_016354.17 (intron 10)). Amplifications were performed in a MyCyclerTM thermal cycler system (Bio‐Rad) and PCR conditions were as following: initial denaturation at 94°C for 1 min, then 35 amplification cycles, denaturation at 94°C for 30 s, annealing at 54°C for 30 s, and chain elongation at 72°C for 1 min. The final extension step was performed at 72°C for 10 min. The PCR products were digested with HincII or EcoRV restriction endonuclease (Thermo) in a 10 µL reaction mixture for 2 hr at 37°C, then electrophoresed on a 2.5% agarose gel and stained with Genecolour fluorescent dye. For rs4693608, the enzyme digestion resulted in an 83bp band and a 41bp band for the A allele and a nondigested 124bp fragment for the G allele. For rs4364254, the T allele was identified by the presence of 226bp fragments and the C allele was represented by 192bp fragments and 34bp fragments. About 10% of the samples were selected randomly to genotype again for quality control, and the concordance rate was 100%.

### 2.4 Statistical analysis

The statistical analyses were carried out using SPSS 22.0 (SPSS, Inc) and SNPstats online software (www.snpstats.net/start.htm). Data were shown as the mean ± standard deviation (*SD*). Differences in variables were evaluated by student's *t* test or *χ* ^2^ test between EC and control groups. Moreover, a chi‐squared analysis was used to determine the allele or genotype frequency differences between cases and controls and to asses Hardy–Weinberg equilibrium. The odds ratios with 95% confidence intervals (CI) were calculated by SNPstats to investigate the effect of SNPs on EC using codominant, dominant, recessive, or overdominant genetic models^23^. *P*‐values less than .05 were considered to be significant.

## 3 RESULTS

### 3.1 Characteristics of the study subjects

The present study included 610 subjects and their clinicopathological features are shown in Table 1. There were no significant differences between the mean age (*p* = .195), BMI (*p* = .294), parity (*p* = .744), family history of cancer (*p* = .296), or menopausal state (*p* = .8) of the two groups. Among all the 270 cases, 202 (74.81%) patients were in FIGO stage I, 95 (35.19%) patients were diagnosed with grade I carcinoma and endometrioid adenocarcinoma ranks first among all pathological type (84.81%).

**Table 1:** Characteristics of EC patients and controls

Characteristics	Patients	Controls	*p* value
Sample size	270	320	
Age(mean ± *SD*) (y)	51.93 ± 9.68	50.84 ± 10.51	.195
BMI(mean ± *SD*) (kg/m^2^)	24.21 ± 3.46	23.93 ± 3.54	.294
Parity(mean ± *SD*)	3.10 ± 1.69	3.06 ± 1.79	.744
Family history of cancer			.296
Yes	20 (7.4%)	17 (5.3%)	
No	250 (92.6%)	303 (94.7%)	
Menopausal state			.8
No	126 (46.7%)	146 (45.6%)	
Yes	144 (53.3%)	174 (54.4%)	
FIGO stage			
I	202 (74.8%)		
II	25 (9.2%)		
III	29 (10.7%)		
IV	13 (4.7%)		
Unknown	2 (0.6%)		
Grade			
I	95 (35.2%)		
II	99 (36.7%)		
III	76 (28.1%)		
Histology			
Endometrioid	229 (84.8%)		
Nonendometrioid	41 (15.2%)		
ER/PR			
Negative	20 (7.4%)		
Positive	204 (75.6%)		
Unknown	46 (17.0%)		

### 3.2 Associations between HPSE gene polymorphisms and risk of EC

Both allelic and genotypic association analyses were carried out. Data were available from 270 cases and 340 controls for statistical analyses and genotype distributions of both rs4693608 and rs4364254 were consistent with the Hardy–Weinberg equilibrium. The genotype and allele frequencies of the two SNPs in both cases and controls are shown in Table 2. For rs4693608, the frequencies of A allele and G allele were 74.0%, 69.0%, and 26.0%, 31.0%, respectively. There existed obvious statistical difference in the genetic frequencies between EC patients and controls. Significant decreased EC risks were found to be correlated with G allele (OR = 0.77, 95%CI = 0.60–0.99, *p* = .04). In the codominant model, the genotype frequencies of AA, GA, and GG for rs4693608 were 47.6%, 41.8%, and 10.6% in the EC group and 52.6%, 42.6%, and 4.8% in the control group. Compared with the genetic type AA, GG showed a protective effect from EC in both codominant (adjusted OR = 0.41, 95%CI = 0.21–0.81, *p* = .026) and recessive models (adjusted OR = 0.43, 95%CI = 0.22–0.82, *p* = .0076). For rs4364254, most of those with the rs4364254 SNP were homozygous for the T/T genotype. However, no significant differences were found in the incidences of EC patients with the rs4364254 polymorphisms compared to controls.

**Table 2:** Genotype and allele distribution of two HPSE polymorphisms in patients with EC and health controls

Genotype or allele	Genotype	Patients	Control	Logistic regression		Logistic regression	
		*N* = 270	*N* = 320	OR (95%CI)	*p* value	OR (95%CI)	*p* value
**rs4693608**							
Genetic model							
Codominant	A/A	162 (47.6%)	142 (52.6%)	1		1	
	G/A	142 (41.8%)	115 (42.6%)	0.92 (0.66–1.29)	.026	0.91(0.65–1.28)	.038
	G/G	36 (10.6%)	13 (4.8%)	0.41 (0.21–0.81)		0.43 (0.21–0.84)	
Dominant	A/A	162 (47.6%)	142 (52.6%)	1	.22	1	.21
	G/A‐G/G	178 (52.4%)	128 (47.4%)	0.82 (0.60–1.13)		0.82 (0.59–1.13)	
Recessive	A/A‐G/A	304 (89.4%)	257 (95.2%)	1	.0076	1	.012
	G/G	36 (10.6%)	13 (4.8%)	0.43 (0.22–0.82)		0.44 (0.23–0.86)	
Overdominant	A/A‐G/G	198 (58.2%)	155 (57.4%)	1	.84	1	.95
	G/A	142 (41.8%)	115 (42.6%)	1.03 (0.75–1.43)		1.01 (0.73–1.40)	
Log‐additive	——	——	——	0.76 (0.59–0.99)	.038	0.77 (0.59–0.99)	.044
Allele							
	A	399 (74.0%)	466 (69.0%)	1	.04		
	G	141 (26.0%)	214 (31.0%)	0.77(0.60–0.99)			
**rs4364254**							
Genetic model	T/T	156 (45.9%)	144 (53.3%)	1	.16	1	.145
Codominant	T/C	152 (44.7%)	101 (37.4%)	0.72 (0.51–1.01)		0.75 (0.53–1.05)	
	C/C	32 (9.4%)	25 (9.3%)	0.85 (0.48–1.50)		0.87 (0.49–1.54)	
Dominant	T/T	156 (45.9%)	144 (53.3%)	1	.067	1	.097
	T/C‐C/C	184 (54.1%)	126 (46.7%)	0.74 (0.54–1.02)		0.77 (0.56–1.06)	
Recessive	T/T‐T/C	308 (90.6%)	245 (90.7%)	1	.95	1	.24
	C/C	32 (9.4%)	25 (9.3%)	0.98 (0.57–1.70)		0.99 (0.57–1.72)	
Overdominant	T/T‐T/C	188 (55.3%)	169 (62.6%)	1	.069	1	.097
	T/C	152 (44.7%)	101 (37.4%)	0.74 (0.53–1.02)		0.76 (0.55–1.06)	
Log‐additive	——	——	——	0.84 (0.65–1.07)	.15		
Allele							
	T	389 (72.0%)	464 (68.0%）	0.83 (0.65–1.07)	.15		
	C	151 (28.0%)	216 (32.0%)				

### 3.3 Association of HPSE gene polymorphisms with clinical characteristics of patients with EC

Tables 3 and 4 showed the stratified analyses between *HPSE* SNPs and clinicopathological parameters. Notably, rs4693608 was associated with tumor grade (*p* = .0023 in codominant model, *p* = .03 in dominant model, *p* = .0016 in recessive model), histology (*p* = .036), and cervical invasion (*p* = .042) in EC patients, and rs4364254 was shown to be associated with tumor grade (*p* = .024 in codominant model, *p* = .009 in overdominant model) alone. No significant association was observed between the two SNPs and other parameters including FIGO stage, myometrial invasion, parametrial invasion, lymph node metastasis, or peritumor intravascular cancer emboli.

**Table 3:** Association between the genotype frequencies of rs4693608 and clinicopathological characteristics of EC patients

Clinical features	Genotype			rs4693608							
				Genetic model							
				Codominant		Dominant		Recessive		Overdominant	
				(A/A vs. G/A vs. G/G)		(A/A vs. G/A‐G/G)		(A/A‐G/A vs. G/G)		(A/A‐G/G vs. G/A)	
	A/A	G/A	G/G	OR(95%CI)	*p* value	OR(95%CI)	*p* value	OR(95%CI)	*p* value	OR(95%CI)	*p* value
FIGO stage											
I	100	89	11	G/A:0.64 (0.36–1.14)	.21	0.62 (0.35–1.09)	.092	0.52 (0.11–2.41)	.37	0.68 (0.38–1.20)	.18
II‐IV	42	24	2	G/G:0.43 (0.09–2.04)							
FIGO grade											
G1	41	43	10	G/A:0.68 (0.40–1.14)	.0023	0.57 (0.34–0.95)	.03	0.15 (0.04–0.55)	.002	0.82 (0.49–1.36)	.44
G2‐G3	100	71	3	G/G:0.12 (0.03–0.47)							
Histology											
Endometrioid adenocarcinoma	119	97	13	G/A:0.96 (0.49–1.88)	.11	0.85 (0.43–1.65)	.63	0.00 (0.00‐NA)	.036	1.06 (0.54–2.08)	.85
Nonendometrioid adenocarcinoma	23	18	0	G/G:0.00 (0.00‐NA)							
Myometrial invasion											
<1/2	105	85	11	G/A:0.87 (0.48–1.58)	.71	0.84 (0.47–1.50)	.55	0.60 (0.13–2.76)	.49	0.91 (0.51–1.64)	.75
≥1/2	34	24	2	G/G:0.56 (0.12–2.66)							
Cervical invasion											
Negative	112	99	12	G/A:0.53 (0.26–1.05)	.11	0.50 (0.26–0.99)	.042	0.40 (0.05–3.15)	.33	0.57 (0.29–1.12)	.095
Positive	30	14	1	G/G:0.31 (0.04–2.49)							
Parametrial invasion											
Negative	127	106	13	G/A:0.56 (0.22–1.42)	.15	0.50 (0.20–1.26)	.13	0.00 (0.00‐NA)	.13	0.62 (0.24–1.57)	.3
Positive	15	7	0	G/G:0.00 (0.00‐NA)							
Lymph node metastasis											
Negative	127	104	13	G/A:0.65 (0.27–1.60)	.19	0.58 (0.24–1.42)	.22	0.00(0.00‐NA)	.12	0.72 (0.29–1.76)	.46
Positive	15	8	0	G/G:0.00 (0.00‐NA)							
Lymphovascular space invasion											
Negative	122	95	12	G/A: 1.28 (0.64–2.56)	.57	1.20 (0.61–2.37）	.6	0.48 (0.06–3.77）	.44	1.34 (0.68–2.65)	.4
Positive	19	19	1	G/G: 0.54 (0.07–4.35)							

**Table 4:** Association between the genotype frequencies of rs4364254 and clinicopathological characteristics of EC patients

Clinical features	rs4364254																		
				Genetic model															
	Genotype			Codominant				Dominant				Recessive				Overdominant			
				(T/T vs. T/C vs. C/C)				(T/T vs. T/C‐C/C)				(T/T‐T/C vs. C/C)				(T/T‐C/C vs. T/C)			
	T/T	T/C	C/C	OR(95%CI)		*p* value		OR(95%CI)		*p* value		OR(95%CI)		*p* value		OR(95%CI)		*p* value	
FIGO stage																			
I	102	80	18	0.65 (0.36–1.19)		.37		0.69 (0.39–1.20)		.18		0.98 (0.37–2.58)		.96		0.67 (0.37–1.21)		.18	
II‐IV	41	21	6	0.83 (0.31–2.24)															
FIGO grade																			
G1	40	45	9		0.47 (0.28–0.81)		.02		0.51 (0.31–0.85)		.09		0.96 (0.41–2.26)		.92		0.50 (0.30–0.84)		.009
G2‐G3	103	55	16		0.69 (0.28–1.69)														
Histology																			
Endometrioid adenocarcinoma	121	89	19		0.71 (0.34–1.50)		.32		0.88 (0.45–1.71)		.7		1.89 (0.71–5.07)		.22		0.65 (0.32–1.34)		.24
Nonendometrioid adenocarcinoma	23	12	6		1.66 (0.60–4.61)														
Myometrial invasion																			
＜1/2	102	81	18		0.63 (0.33–1.19)		.34		0.69 (0.38–1.23)		.21		1.13 (0.43–2.99)		.81		0.63 (0.34–1.18)		.14
≥1/2	36	18	6		0.94 (0.35–2.56)														
Cervical invasion																			
Negative	114	87	22		0.63 (0.32–1.27)		.19		0.58 (0.30–1.12)		.1		0.42 (0.10–1.87)		.21		0.71 (0.36–1.40)		.31
Positive	29	14	2		0.36 (0.08–1.61)														
Parametrial invasion																			
Negative	132	94	20		0.89 (0.33–2.39)		.35		1.16 (0.48–2.77)		.74		2.51 (0.77–8.14)		.15		0.75 (0.30–1.92)		.55
Positive	11	7	4		2.40 (0.70–8.27)														
Lymph node metastasis																			
Negative	128	96	20		0.48 (0.17–1.37)		.14		0.71 (0.30–1.70)		.44		2.36 (0.73–7.61)		.18		0.43 (0.15–1.19)		.084
Positive	14	5	4		1.83 (0.55–6.11)														
Peritumor intravascular cancer emboli																			
Negative	119	88	22		0.76 (0.37–1.59)		.71		0.75 (0.38–1.50)		.42		0.78 (0.22–2.76)		.7		0.80 (0.39–1.64)		.54
Positive	23	13	3		0.71 (0.19–2.55)														

## 4 DISCUSSION

Upregulation of *HPSE* is detected in a wide range of human cancers by immunohistochemistry, in situ hybridization, real‐time PCR analyses and is shown to correlate with metastatic potentials (Barash et al., 2010). In EC, previous studies showed higher *HPSE* expression in endometrial carcinoma of grade 2 + 3, advanced FIGO stage and carcinoma with deep myometrial invasion, positive lymph node, lymphvascular space involvement (Canaani et al., 2008; Inamine et al., 2008; Hasengaowa et al., 2006). Hasengaowa et al indicated deteriorating prognoses (both disease‐free and overall survival) of 166 EC patients associated with elevated *HPSE* expression levels (Hasengaowa et al., 2006). The study of Watanabe et al found a strong association between *HPSE* and microvessel density, suggesting its important role in promoting tumor angiogenesis.

Genetic variation has been known to influence gene regulation and contribute to disease risk in variable ways. Huang et al demonstrated a close relationship of allele loss and reduced *HPSE* expression with tumor progression and poor prognosis in hepatocellular carcinoma (Huang et al., 2012). The study of Ostrovsky et al also demonstrated a relationship between certain SNPs with *HPSE* expression level and proposed a possible mechanism of self‐regulation in a SNP‐dependent manner (Ostrovsky et al., 2018). However, the functional role of *HPSE* SNPs in EC risk and in the regulation of its gene expression has not been elucidated. This is perhaps the first study that evaluated the role of *HPSE* SNPs in EC.

The SNPs of rs4693608 and rs4364254 were both located at introns, mapping in nucleotide position 8,736,062 and nucleotide position 8,718,418, respectively. In the present study, we analyzed the associations between the two *HPSE* SNPs and EC risk as well as certain clinical features using logistic regression analysis. The data revealed statistically significant differences in the distributions of both *HPSE* genotypes and alleles. For rs4693608, logistic regression analysis indicated that A/A promotes susceptibility to EC significantly, which is in line with previous studies. Moreover, a significantly increased distribution of A/A was observed in patients with grade ≥ 2 (*p* = .03) and in patients with positive cervical invasion (*p* = .042), and the G/G genotype displayed a remarkably decreased distribution in patients with grade ≥ 2 (*p* = .0016). For rs4364254, the results revealed that patients with T/C genotype had lower tumor grade than subjects with TT or CC genotypes.

Previous studies exploring the role of *HPSE* polymorphisms in diverse diseases reported variable results. Andersen et al evaluated the relationships of four *HPSE* SNPs with multiple myeloma patients and found that the rs4693608 genotype A/A increased the susceptibility to vertebral fractures significantly, which may be result from the higher *HPSE* mRNA expression in carriers of the rs4639608 A/A that stimulates osteoclastogenesis and osteoclast activity through RANKL activation and inhibiting osteoblastogenesis (Andersen et al., 2015). Ostrovsky, Shimoni, Rand, Vlodavsky, & Nagler, 2010 reported an increased risk of acute graft‐versus‐host disease and significantly different *HPSE* expression level in patients with A/A (rs4693608) and T/T (rs4364254) genotypes (Ostrovsky et al., 2010). As both the two SNPs are located in the intronic region, they proposed that this difference may be caused by the regulation effect of their carrying sequence which can modify DNA‐protein interactions. Seifert C demonstrated similar results in sinusoid obstruction syndrome patients (Seifert, Wittig, Arndt, & Gruhn, 2015). No statistically significant differences of the allele frequencies and genotypic frequencies of rs4693608 and rs4364254 were found between patients and cancer‐free controls in gastric cancer or hematological malignancies(Ostrovsky et al., 2010; Seifert et al., 2015). However, Li et al found that both A/A (rs4693608) and T/T (rs4364254) had prognostic value for gastric‐specific survival, which is in accordance with ours revealing that the two genotypes predicted tumor grade and cervical invasion (Li et al., 2012; Yue et al., 2010). They attributes this difference to the relatively high mRNA level of A/A (rs4693608) and T/T (rs4364254), which is similar to the mechanism proposed by Ostrovsky et al (Ostrovsky et al., 2007).

However, there are a few limitations that should be taken into consideration. A total of 590 patients may not be evident enough to identify the role of *HPSE* in EC. Moreover, although *HPSE* SNPs are shown to be risk factors for EC, the latent diseases among the population may cause relatively great heterogeneity. Additionally, the association of *HPSE* expression and SNPs as well as related molecular mechanism are needed to be substantiated further. These limitations should be noted.

In conclusion, the results of our present study demonstrated a strong association between *HPSE* SNPs and EC, suggesting an important role of *HPSE* in modulating EC carcinogenesis. Our analyses showed that genotypic frequencies as obtained from the codominant and recessive genetic models for rs4693608 correlated with susceptibility to EC. However, a larger sample size and more evidence are needed to support the early observations of this study.

## CONFLICT OF INTEREST

The authors declare that they have no conflict of interest.

## AUTHORS’ CONTRIBUTION

HYC and SY conceived and designed the experiments. HYC and MRX performed the experiments. MRX supervised the experiments. HYC, SY, and XZY analyzed the data. MRX provided study patients. HYC wrote the manuscript. MRX revised the manuscript. All listed authors approved the final version of the manuscript.
